# Cerebral Blood Flow Hemispheric Asymmetry in Comatose Adults Receiving Extracorporeal Membrane Oxygenation

**DOI:** 10.3389/fnins.2022.858404

**Published:** 2022-04-11

**Authors:** Thomas W. Johnson, Irfaan A. Dar, Kelly L. Donohue, Yama Y. Xu, Esmeralda Santiago, Olga Selioutski, Mark A. Marinescu, Ross K. Maddox, Tong Tong Wu, Giovanni Schifitto, Igor Gosev, Regine Choe, Imad R. Khan

**Affiliations:** ^1^Department of Neurology, University of Rochester Medical Center, Rochester, NY, United States; ^2^Department of Biomedical Engineering, University of Rochester, Rochester, NY, United States; ^3^Department of Neurology, Northwestern University Feinberg School of Medicine, Chicago, IL, United States; ^4^School of Arts and Sciences, University of Rochester, Rochester, NY, United States; ^5^Department of Medicine, University of Rochester Medical Center, Rochester, NY, United States; ^6^Department of Neuroscience, University of Rochester Medical Center, Rochester, NY, United States; ^7^Department of Biostatistics and Computational Biology, University of Rochester, Rochester, NY, United States; ^8^Division of Cardiac Surgery, Department of Surgery, University of Rochester Medical Center, Rochester, NY, United States; ^9^Department of Electrical and Computer Engineering, University of Rochester, Rochester, NY, United States

**Keywords:** cerebrovascular autoregulation, coma, diffuse correlation spectroscopy, extracorporeal membrane oxygenation, cerebral blood flow

## Abstract

Peripheral veno-arterial extracorporeal membrane oxygenation (ECMO) artificially oxygenates and circulates blood retrograde from the femoral artery, potentially exposing the brain to asymmetric perfusion. Though ECMO patients frequently experience brain injury, neurologic exams and imaging are difficult to obtain. Diffuse correlation spectroscopy (DCS) non-invasively measures relative cerebral blood flow (rBF) at the bedside using an optical probe on each side of the forehead. In this study we observed interhemispheric rBF differences in response to mean arterial pressure (MAP) changes in adult ECMO recipients. We recruited 13 subjects aged 21–78 years (7 with cardiac arrest, 4 with acute heart failure, and 2 with acute respiratory distress syndrome). They were dichotomized *via* Glasgow Coma Scale Motor score (GCS-M) into comatose (GCS-M ≤ 4; *n* = 4) and non-comatose (GCS-M > 4; *n* = 9) groups. Comatose patients had greater interhemispheric rBF asymmetry (ASYM_rBF_) vs. non-comatose patients over a range of MAP values (29 vs. 11%, *p* = 0.009). ASYM_rBF_ in comatose patients resolved near a MAP range of 70–80 mmHg, while rBF remained symmetric through a wider MAP range in non-comatose patients. Correlations between post-oxygenator pCO_2_ or pH vs. ASYM_rBF_ were significantly different between comatose and non-comatose groups. Our findings indicate that comatose patients are more likely to have asymmetric cerebral perfusion.

## Introduction

Extracorporeal membrane oxygenation (ECMO) is a method of external mechanical circulatory support (MCS) designed to oxygenate and circulate blood, thus supplementing insufficient respiratory and cardiac functions during critical illness. Its use has increased in the preceding decade as technology and techniques have improved. However, approximately 13% of ECMO recipients are found to have neurologic injuries either from underlying pathology or the ECMO therapy itself (Migdady et al., 2019; Shoskes et al., 2020; Kietaibl et al., 2021). Performing accurate neurologic examinations in this population is challenging due to sedation and neuromuscular blockade (NMB) requirements to prevent cannula dislodgement, optimize hemodynamics, or to force ventilator compliance (Debacker et al., 2018; Patel et al., 2020). Neuroimaging requires resource-intensive, multi-disciplinary transportation to imaging suites (Prodhan et al., 2010), while invasive neuromonitoring can further increase the risk of cerebral hemorrhage due to anticoagulation requirements while on ECMO (Thomas et al., 2018). Thus, the brain’s perfusion and oxygenation requirements are not routinely monitored in current clinical practice, furthering the potential for injury.

Diffuse correlation spectroscopy (DCS) provides non-invasive, continuous bedside neuromonitoring using near-infrared light, modeled with the correlation diffusion equation (Durduran et al., 2010a), to measure blood flow index (BFI), which is directly proportional to cerebral blood flow (CBF) (Durduran and Yodh, 2014). Its use to monitor CBF has been described in a number of brain-injured cohorts (Durduran et al., 2009; Delgado-Mederos et al., 2018; Baker et al., 2019) and has been validated against other CBF measurement modalities including transcranial Doppler (TCD) (Durduran et al., 2010b; Kim et al., 2010; Mesquita et al., 2011). DCS is closely related to near-infrared spectroscopy (NIRS), a commercially available technology that measures cerebral oxygenation, but DCS offers the advantage of measuring CBF directly rather than using oximetry as a surrogate marker.

Veno-arterial (VA) ECMO is often configured to supply oxygenated blood in retrograde flow up the aorta *via* a cannula placed in the femoral artery. This is contrasted with veno-venous (VV) ECMO, in which oxygenated blood is returned to the body *via* the venous system and perfuses the arterial system in a normal physiologic manner. This distinction is important as asymmetric cerebral and somatic oxygenation and perfusion have been described in VA ECMO (Cove, 2015; Acharya et al., 2017; Nezami et al., 2021). The presence of disrupted cerebrovascular autoregulation in comatose patients with hypoxic brain injury furthers the possibility of asymmetric perfusion arising from the brain’s interaction with the ECMO circuit and the unique changes in circulatory physiology ECMO produces (Van Den Brule et al., 2018). We used DCS to record hemispheric CBF in adult ECMO patients with the hypothesis that comatose patients will exhibit more interhemispheric asymmetry than in non-comatose patients.

## Materials and Methods

### Patient Recruitment

This prospective cohort study was approved by the Institutional Review Board (IRB) at the University of Rochester Medical Center. Adult patients aged 18 years and older admitted to the cardiac intensive care unit (CICU) for either VA or VV ECMO therapy irrespective of etiology at the University of Rochester Medical Center (URMC) between 12/2019 and 7/2021 were eligible for participation. Informed consent was obtained from patients’ legally authorized representatives 24 h after ECMO initiation. Exclusion criteria consisted of pre-existing neurologic conditions and facial injuries impeding DCS measurement.

### Experimental Design

A detailed description of the experimental design and instrumentation used for this study has been previously published (Dar et al., 2020). In summary, enrolled patients underwent a protocol of daily CBF monitoring using an in-house designed DCS system as well as a commercially available TCD system. Both devices were approved by URMC’s Clinical Engineering Department. The DCS system employs a 785 nm laser with long coherence length to non-invasively quantify BFI, which is proportional to CBF (Carp et al., 2010; Durduran et al., 2010b; Kim et al., 2010; Mesquita et al., 2011). Two slim-profiled optical probes using a 45-degree prism were attached to the left and right forehead of the patient using double-sided tape (3M, St. Paul, MN, United States) and Tegaderm (3M, St. Paul, MN, United States). Light emitted from these two probes propagated into the tissue, then was collected by single mode optical fibers at 2.5 cm separation from the source that were connected to single-photon counting detectors. Autocorrelation curves were calculated using a 2 s integration time, which translated to BFI values at 0.25 Hz for each hemisphere. BFI values were calculated from the autocorrelation curves using constant absorption and scattering coefficients previously reported in the literature for brain measurements (Brady et al., 2010; Parthasarathy et al., 2018; Selb et al., 2018; Dar et al., 2020). Data were discarded if artifacts were present or had poor signal to noise ratio (SNR), which was defined as less than 4 kilo-counts per second (kcps) measured by the detectors.

Transcranial Doppler was performed on subjects at the bedside using the ST3 system (Spencer Technologies, Redmond, WA, United States). Cerebral blood flow velocity (CBFV) was measured from both middle cerebral arteries, one at a time, for up to 5 min on each day during simultaneous DCS recording. Measurements were performed once daily using a single TCD probe held at the temporal window by trained study staff. Mean CBFV was reported at 1 Hz frequency, and data were discarded if the SNR was low or artifacts were present.

Continuous physiological data including systolic, diastolic, and mean arterial pressure (MAP) were recorded continuously at the bedside using MediCollector software (MediCollector Inc., Boston, MA, United States) at 1 Hz. ECMO pump speed and flow were also recorded at 0.2 Hz from the ECMO machine. BFI and physiologic monitoring were performed for up to 3 h during the initial resuscitation phase, during which ECMO fully supported the circulation at constant pump speeds. Monitoring was performed for up to 8 h during the ECMO wean phase while pump speeds were gradually tapered by clinical staff to test native cardiovascular function. This prolonged duration was performed to capture as much hemodynamic change as possible.

### Cerebral Autoregulation Asymmetry Analysis

**FIGURE 1:**
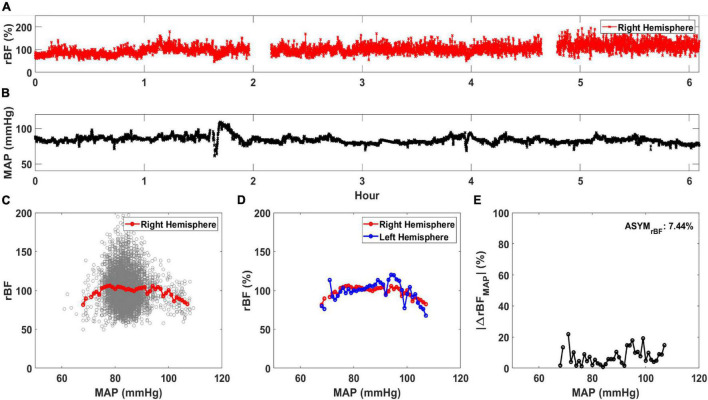
Calculation of average relative blood flow (rBF, in %) vs. mean arterial pressure (MAP). An example time trace for **(A)** rBF and **(B)** MAP for a single day of monitoring are shown. Using these time traces, **(C)** rBF is plotted against MAP and an average rBF value at each MAP is calculated (red circle). **(D)** An averaged rBF vs. MAP curve is generated for both the right (red circle) and left hemisphere (blue circle). Using the data in **(D)**, |ΔrBF_MAP_| (in %/mmHg) is plotted against MAP in **(E)**. ASYM_rBF_, averaged |ΔrBF_MAP_| of this monitoring day for a patient is shown on the upper right corner.

Cerebral autoregulation curves correlating cerebral perfusion with MAP were created from left and right hemispheric DCS data for all patients. All analysis was performed using MATLAB 2020 (Mathworks, Natick, MA, United States). BFI and MAP data were first resampled to 0.25 Hz to ensure a uniform time series between the data sets. The continuous measurement of relative blood flow (rBF, in %) was determined for each day of DCS monitoring to evaluate the relative change in BFI for all subjects. This was calculated by normalizing the BFI data of each hemisphere by the median BFI value of that hemisphere (Selb et al., 2018). Figure 1A displays the rBF for the right hemisphere of one subject while Figure 1B shows the concurrent MAP. Using the concurrent rBF and MAP time series, rBF values were plotted against MAP values between 50 and 120 mmHg at 1 mmHg increments shown in the background scatterplot of Figure 1C. From this scatterplot, the average rBF value was calculated at each MAP value, shown as the red circles in Figure 1C. Average rBF values were discarded if there were less than 5 data points at the specific MAP value. Figure 1D shows the average curve for both left and right hemisphere.

Using these data, asymmetry between the hemispheric data was calculated for each day of monitoring for all subjects. First, the magnitude of difference was calculated as |ΔrBF_MAP_(MAP_i_)| defined in Equation 1 and reported as %. MAP_i_ are the MAP values in increments of 1 mmHg derived from the MAP range that day, to a maximum MAP value at MAP_N_, where N is the total number of MAP increments of the monitoring day. We averaged |ΔrBF_MAP_(MAP_i_)| to calculate ASYM_rBF_ for the monitoring day, shown in Equation 2 and Figure 1E, reported as %.


(1) $$\left|\Delta \,{\text{rBF}}_{\text{MAP}}\,\left({\text{MAP}}_{\text{i}}\right)\right|=\left|{\text{rBF}}_{\text{left}}\,\left({\text{MAP}}_{\text{i}}\right)-{\text{rBF}}_{\text{right}}\,\left({\text{MAP}}_{\text{i}}\right)\right|,\;i=1,2,\ldots \,\text{N}$$

(2) $${\text{ASYM}}_{\text{rBF}}=\frac{1}{\text{N}}\,\sum_{\text{i}=1}^{\text{N}}\left|\Delta \,{\text{rBF}}_{\text{MAP}}\,\left({\text{MAP}}_{\text{i}}\right)\right|,i=1,2,\ldots \,\text{N}$$

TCD data were used to calculate the absolute interhemispheric difference in relative CBFV, | ΔrCBFV_MAP_(MAP_i_)|, similar to above and shown in Equation 3.


(3) $$|\Delta {\text{rCBFV}}_{\text{MAP}}\left({\text{MAP}}_{\text{i}}\right)|=|{\text{rCBFV}}_{\text{left}}\left({\text{MAP}}_{\text{i}}\right)-{\text{rCBFV}}_{\text{right}}\left({\text{MAP}}_{\text{i}}\right)|,i=1,2,\ldots \text{N}$$

rCBFV was determined using the same method as the rBF calculation, which was to divide the measured CBFV by its median value. These values were used to compare asymmetry measured by TCD with asymmetry measured by DCS (Altman and Bland, 1983; Giavarina, 2015).

### Clinical Characteristics

Data obtained from the electronic medical record data for each patient were recorded in a secure REDCap database. We recorded demographics, ECMO characteristics (duration, location of cannula, and reason for cannulation), and daily clinical characteristics. Clinical characteristics included Glasgow Coma Scale (GCS), Sequential Organ Failure Assessment (SOFA), arterial blood gas (ABG) parameters including pH, partial pressure of arterial oxygen (pO_2_), partial pressure of arterial CO_2_ (pCO_2_), lactate, cardiac left ventricular ejection fraction (LVEF), and sedation/analgesia infusion rates.

### Cerebral Autoregulation Asymmetry vs. Comatose Status

Per institutional standard of care, clinical nursing staff neurologically examined each subject and documented the GCS. Examinations were performed at least once daily when clinically feasible (e.g., without significant risk of ventilator dyssynchrony or cannula dislodgement) with sedation and NMB pauses. When sedation/NMB was unable to be paused, the GCS was documented as is with infusion running. For this study, each subject’s highest daily neurologic GCS Motor (GCS-M) subscores were recorded during monitoring (see Supplementary Table 1). Subjects who were able to attain a GCS-M score of 6 at any time during ECMO were characterized as “non-comatose,” while those who were remained unconscious with GCS-M ≤ 4 were categorized as “comatose.” This definition was based on prior studies of cardiac arrest patients where therapeutic hypothermia was initiated in comatose patients who were unconscious and unable to follow verbal commands (Dankiewicz et al., 2021). Since all subjects in our study that had GCS-M scores of 5 eventually reached a score of 6, we chose GCS-M 4 as the dichotomization cut-off. To evaluate the difference in ASYM_rBF_ between comatose and non-comatose patients, the maximum value of ASYM_rBF_ from the monitoring period was determined.

### Statistical Analysis

One-tailed and two-tailed two-sample *t*-tests were used to compare maximum ASYM_rBF_ in comatose vs. non-comatose patients. Normality was checked using the Shapiro–Wilks test, and variances between the two groups were tested using a *F*-test. Alternative hypotheses were defined for both the one-tailed and two-tailed test. With the *F*-test result and appropriate alternative hypothesis, *p*-values were calculated for both the one-tailed and two-tailed *t*-test. To compare data collection methodologies, TCD and DCS blood flow asymmetry measurements were compared using Bland–Altman analysis over the range of MAP values the patient experienced during simultaneous TCD and DCS recording (Altman and Bland, 1983; Giavarina, 2015). The average difference per patient was calculated between |ΔrBF_MAP_(MAP_i_)| and |ΔrCBFV_MAP_(MAP_i_)|, and a two-sided one-sample rank-sum test was performed after normality test revealed a non-normal distribution, and a *p*-value was calculated. The *p*-values were compared to a significance level α of 0.05, and was considered statistically significant if less than α. Scatter plots with Pearson correlation coefficients were calculated to assess whether pCO_2_, pO_2_, pH, and LVEF associated with either maximal or daily ASYM_rBF_ for each patient. Statistical analysis was performed using MATLAB software.

## Results

Out of 62 of eligible patients undergoing ECMO treatment for different indications, thirteen (21%) were consented for participation (five females; mean age 46 years [range 21–78]). Patient demographics and pertinent clinical parameters are shown in Table 1. Seven subjects required ECMO for cardiac arrest, four for cardiogenic shock and two for acute respiratory distress syndrome (ARDS). VA ECMO was performed on 11 patients and VV ECMO on the two patients with ARDS. The mean length of hospital stay was 27 days. The mean duration of ECMO treatment was 266 h. Mean pre-ECMO SOFA score was 12. Nine subjects (69%) were classified as “awake” and four (31%) as “comatose” as defined above. Nine subjects survived to ECMO decannulation and seven to hospital discharge. A total of 63 monitoring sessions were conducted, with an average of five sessions per subject. Data from seven sessions were omitted from analysis due to low SNR (subject 3, days 1 through 6 and subject 11, day 3). Data for the first 6 days of Subject 3’s recording were discarded due to jaundice, which confounded DCS signal strength. Subject 11 had low signal quality that could not be corrected from the right hemisphere on day 3 which was also discarded.

**TABLE 1:** Patient demographics and pertinent clinical data.

Subject	Best GCS-M	Coma	Age range	Sex	ECMO duration (hours)	LOS (days)	ECMO indication	ECMO type	Cannula location	Pre-ECMO SOFA	Survival to discharge	Cause of death
8	4	Y	61–65	M	131.6	21	CS	VA	FA	13	N	Cardiovascular
9	1	Y	61–65	M	139.1	23	CA	VA	FF	11	N	Sepsis
11	4	Y	21–25	F	304.1	54	CA	VA	FF	13	Y	N/A
13	1	Y	56–60	M	171.7	7	CA	VA	FF	12	N	Cardiovascular
1	6	N	71–75	F	93.2	40	CA	VA	FF	14	Y	N/A
2	6	N	76–80	F	433.6	10	CS	VA	FF	10	N	Sepsis
3	6	N	36–40	M	321.0	19	CS	VA	FF	15	N	Hemorrhagic shock
4	6	N	26–30	F	159.2	26	CS	VA	FF	12	Y	N/A
5	6	N	51–55	M	240.7	66	CA	VA	FF	9	Y	N/A
6	6	N	71–75	M	90.3	33	CA	VA	FF	12	Y	N/A
7	6	N	26–30	M	211.8	42	ARDS	VV	FJ	16	Y	N/A
10	6	N	21–25	M	817.1	84	ARDS	VVV^1^	FFJ	12	Y	N/A
12	6	N	41–45	F	345.7	17	CA	VA	FF	13	N	Cardiovascular

*Shaded rows indicate patients that were defined as comatose.*

*LOS, length of stay; SOFA, Sequential Organ Failure Assessment; ARDS, acute respiratory distress syndrome; CA, cardiac arrest; CS, cardiogenic shock; VA, venoarterial; VV, venovenous; GCS-M, Glasgow Coma Scale Motor score; N/A, not applicable; FA, femoral-axillary; FF, femoral-femoral; FJ, femoral-jugular.*

*^1^Two cannula (femoral and jugular) returning oxygenated blood to patient from two separate oxygenators.*

Daily GCS-M scores recorded during ECMO treatment for each subject are shown in Supplementary Figure 1. Subjects 1, 2, and 4 were awake and remained so, while subjects 8, 9, 11, and 13 remained comatose throughout monitoring. GCS-M scores for subjects 3, 5, 6, 7, 10, and 12 fluctuated but were able to improve to follow commands at some point (GCS-M = 6). Notably, subjects 7 and 10 awoke to follow commands after paralytics and sedation were weaned, while the exam for subject 5 improved spontaneously on day 4.

Table 2 details the clinical characteristics for all subjects. These include cumulative sedation the subjects received during the monitoring period, ABG results, and ECMO circuit pCO_2_ measured both pre and post-oxygenator on the day of maximum observed ASYM_rBF_. Supplementary Table 1 shows the mean values of these clinical characteristics for the comatose and non-comatose groups. None of the values were significantly different between the two groups.

**TABLE 2:** Clinical characteristics of all patients for the day of maximum ASYM_rBF_.

Subject	Monitoring duration (hours)	Cumulative sedation					ABG		ECMO circuitry	
		Fentanyl (mcg/kg)	Dilaudid (mg/kg)	Precedex (mcg/kg)	Propofol (mcg/kg)	Versed (mg/kg)	Lactate (mmol/L)	pCO_2_ (mmHg)	Pre-oxy pCO_2_ (mmHg)	Post-oxy pCO_2_ (mmHg)
8	2.38	229.2	0	2.4	0	0	1.5	31	61	50
9	2.45	183.8	0	0	0	9.8	3.9	28	39	29
11	1.32	230.4	0	0	52.7	0	1.2	40	43	35
13	2.12	211.7	0	1.3	0	0	1.1	43	53	47
1	6.12	359.2	0	0	0	0	3.3	33	34	26
2	2.65	0	0	0	0	0	1.3	36	50	45
3	2.17	0	3.3	1.7	32.5	0	2.3	35	41	36
4	1.60	0	0	0	0	0	1.1	34	41	36
5	4.30	716.3	0	3.0	0	0	1.3	39	47	41
6	2.73	267.9	0	1.4	0	0	2.0	33	28	35
7	1.82	26.3	0	0	0	3.6	1.5	25	36	36
10	2.08	0	14.8	3.1	0	18.2	0.7	67	67	57
12	1.82	0	0	0	0	0	0.4	36	49	41

*Sedation values are the cumulative amount during monitoring period. Shaded rows indicate patients that were defined as comatose. ABG, arterial blood gas. Pre- and post-oxy indicate blood drawn from pre and post oxygenator of the ECMO machine.*

**FIGURE 2:**
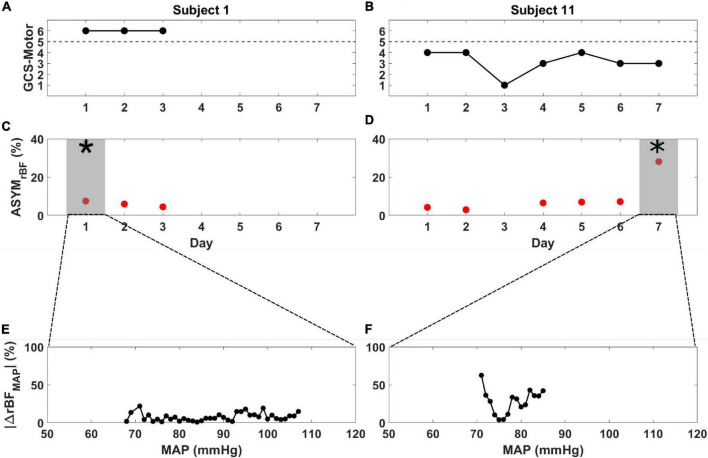
Glasgow Coma Scale Motor score (black), ASYM_rBF_ (red), and |ΔrBF_MAP_| vs. MAP from the day of maximum ASYMrBF for subject 1 **(A,C,E)** and subject 11 **(B,D,F)** during their monitoring periods. **(A)** Subject 1, defined as non-comatose, displayed a constant GCS-Motor score of 6 while **(B)** subject 11, defined as comatose, varied between 1 and 4. ASYM_rBF_ data is shown for **(C)** subject 1 and **(D)** subject 11. The shaded region marked by the asterisk (*) denotes the maximum ASYM_rBF_ value that was chosen for each subject. | ΔrBF_MAP_| of the gray shaded region shown in **(C,D)** is shown for **(E)** subject 1 and **(F)** subject 11. BFI data for day 3 of subject 11 was discarded due to low SNR thus ASYM_rBF_ was not calculated.

**FIGURE 3:**
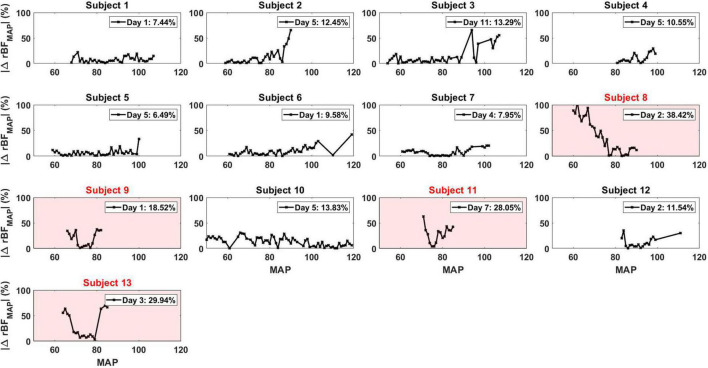
|ΔrBF_MAP_| for all the patients for each specific day that corresponded to their maximum ASYM_rBF_ value. The number displayed in the legend of each plot was the ASYM_rBF_ for that day. Subjects 8, 9, 11, and 13, highlighted in red, were subjects defined to be comatose.

**FIGURE 4:**
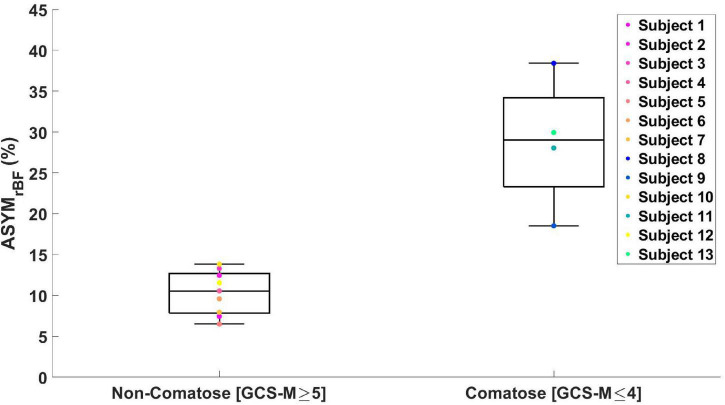
Boxplot of maximum ASYM_rBF_ values for all the subjects grouped by GCS-Motor score. Non-comatose patients had a median value of 11% [IQR 8–13%] and comatose patients 29% [IQR 23–34%] (one-tailed *p* = 0.009, two-tailed *p* = 0.018).

As archetypes of the observed asymmetry phenomenon, Figure 2 highlights ASYM_rBF_ vs. GCS-M data for subjects 1 (awake, conversant) and 11 (comatose, severely brain injured due to cardiac arrest with hypoxic-ischemic encephalopathy confirmed by CT and MRI neuroimaging). Daily ASYM_rBF_ is shown for all subjects in Supplementary Figure 2. For each subject’s day of maximum ASYM_rBF_, Supplementary Figure 3 shows correlation between hemispheric rBF and MAP and Figure 3 displays |ΔrBF_MAP_| . Figure 4 summarizes these data with a group-level comparison: comatose subjects experienced significantly higher ASYM_rBF_ values compared to awake subjects (29% [IQR 23–34%] vs. 11% [IQR 8–13%], one-tailed *p* = 0.009, two-tailed *p* = 0.018).

Time-courses of ΔrBF_MAP_ during the total monitoring period on the day of maximum asymmetry are shown for all patients in Supplementary Figure 4. Among comatose patients, subject 8 showed rBF skewed toward the right hemisphere at lower MAP values while subject 9, 11, and 13 showed rBF values skewed toward the left hemisphere. However, these patients showed equal rBF of both hemispheres at around a MAP value of 80 mmHg. On the contrary, non-comatose patients show an equal rBF throughout the entire MAP range compared to the comatose patients.

**FIGURE 5:**
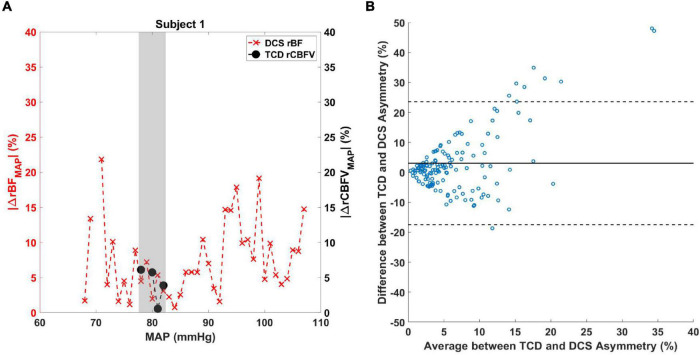
**(A)** Comparison between TCD and DCS measurements of cerebral blood flow asymmetry. |ΔrBF_MAP_(MAP_i_)| (red X marks) and |ΔrCBFV_MAP_(MAP_i_)| (black circles) data are shown for subject 1 over a range of MAP values. The shaded area denotes the MAP values where |ΔrBF_MAP_(MAP_i_)| and |ΔrCBFV_MAP_(MAP_i_)| overlap. **(B)** Bland–Altman analysis was performed to compare the difference between the two measurements against the average of the two measurements. Solid line indicates the mean of the *y*-axis and dashed lines are the 95% confidence interval.

Regarding TCD and DCS comparison analysis, an example of the difference in MAP range experienced by patients is illustrated in Figure 5A. Due to the limitations in obtaining continuous TCD measurements in the MAP range, only values over a limited MAP range were acquired compared to DCS. The shaded region denotes the overlap between the asymmetry quantified by DCS and TCD values for subject 1. A Bland–Altman plot comparing |ΔrBF_MAP_| and |ΔrCBFV_MAP_| for all patient data is shown in Figure 5B, revealing most data to fall within the 95% confidence interval. A non-parametric Wilcox rank sum test resulted in a *p*-value of 0.54, indicating that the two methods do not significantly differ in their measurement of CBF asymmetry. A boxplot showing the individual subjects’ average difference between DCS and TCD asymmetry is shown in Supplementary Figure 5.

**FIGURE 6:**
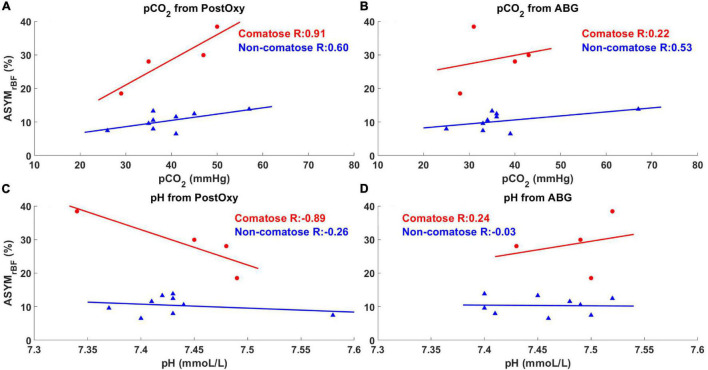
Maximum ASYM_rBF_ vs. pCO_2_ and pH values from post-oxygenator and arterial blood gas (ABG). Red circles indicate comatose subjects and blue triangles indicate non-comatose subjects. pCO_2_ and pH values taken from the post-oxygenator are shown in **(A,C)**, while values taken from the ABG are shown in **(B,D)**. Comatose patients showed higher *R*-value between maximum ASYM_rBF_ and clinical markers **(A,C)** taken from the post-oxygenator compared to non-comatose patients.

Univariate linear modeling with Pearson correlation analysis was performed with ASYM_rBF_ vs. ABG and post-oxygenator pH, pCO_2_, and pO_2_ values, as well as LVEF. Subsets of these analyses are shown in Figure 6. There were no significant associations between ASYM_rBF_ and pO_2_ or LVEF in either comatose or non-comatose groups and are not shown. Neither were any associations found to be significant for ABG data: ASYM_rBF_ vs. ABG pCO_2_, comatose slope = 0.26 %/mmHg (*R* = 0.22, *p* = 0.78), non-comatose slope = 0.12%/mmHg (*R* = 0.53, *p* = 0.15); ASYM_rBF_ vs. ABG pH, comatose slope = 51.04%/(mmol/L) (*R* = 0.24, *p* = 0.76), non-comatose slope = −2.00%/(mmol/L) (*R* = −0.03, *p* = 0.93). In addition, there was no significant association found for post-oxygenator data although excellent *R* values were noted for some cases: ASYM_rBF_ vs. post-oxygenator pCO_2_, comatose slope = 0.75%/mmHg (*R* = 0.91, *p* = 0.09), non-comatose slope = 0.19%/mmHg (*R* = 0.60, *p* = 0.09); ASYM_rBF_ vs. post-oxygenator pH, comatose slope = −105.27%/(mmol/L) (*R* = −0.89, *p* = 0.11), non-comatose slope = −11.87%/(mmol/L) (*R* = −0.26, *p* = 0.50). However, comparison of linear model slopes between the comatose and non-comatose subgroups did reveal a significant difference both for post-oxygenator pCO_2_ (*p* = 0.03) and post-oxygenator pH (*p* = 0.04).

Two of four comatose subjects did not survive to ECMO decannulation and underwent withdrawal of care due to septic shock (subject 9) or severe neurologic injury (subject 13). Subject 8 survived decannulation and regained consciousness, but had care withdrawn in context of subsequent recurrent cardiogenic shock. Subject 11 survived decannulation, remained comatose and was discharged to a coma rehabilitation program but died from recurrent cardiac arrest within 1 week of discharge. Three of the nine non-comatose subjects died prior to discharge due to septic shock (subject 2), hemorrhagic shock (subject 3), or recurrent cardiogenic shock (subject 12).

## Discussion

In this pilot study of 13 patients undergoing ECMO, those who remained comatose throughout their duration of treatment were found to have relatively high hemispheric asymmetry in rBF over a range of blood pressures. In these four subjects, the degree of asymmetry appears to change with MAP, but rBF was elevated on the left hemisphere in 3 (75%) of them. Though ASYM_rBF_ was found to be elevated in comatose subjects, this metric did not associate with survival to discharge. To our knowledge, this is the first study to demonstrate this phenomenon using continuous, non-invasive CBF monitoring *via* DCS.

Multiple possible physiologic reasons may explain the presence of asymmetric perfusion, though our study was not powered to elucidate specific justifications of this phenomenon. All four comatose subjects underwent peripheral VA ECMO, whereby blood is ejected from the ECMO circuit directly into arterial circulation retrograde up the aorta from the femoral artery. Under the pressure of ECMO device, the blood flows against intrinsic cardiac outflow in a mixing cloud (Alwardt et al., 2013). Increasing cardiac contractility pushes this cloud distally, supplying the brain with native cardiac blood *via* the carotid arteries which diverge from the proximal aorta. Conversely, increasing ECMO flow pushes the cloud proximally toward the heart, perfusing the brain with ECMO circulation. Native and ECMO blood typically have different pH levels, oxygen saturations, and amounts of dissolved carbon dioxide. If the mixing cloud lies in between the brachiocephalic trunk and left common carotid artery, the two sides of the brain may receive blood under different pressures and with different levels of oxygen, CO_2_, pulsatility, and pressure (Stevens et al., 2017; Nezami et al., 2021). We did not find significant differences in comatose vs. non-comatose patients in average pH, pCO_2_ from multiple sources, ECMO flows, MAP range, or pulsatility (Supplementary Table 1). Comatose subjects did experience a lower range of pulse pressures, but this did not correlate with ASYM_rBF_ and may have been because they were unconscious and less responsive to external stimuli. A previous study looking at angiography of the brain in VA ECMO patients illustrated this asymmetric blood flow *via* filling time of injected iodine contrast (Acharya et al., 2017). Thus, it is possible that the left hemisphere receives a higher proportion of blood from the ECMO circuit, and at higher flow rates, than the right hemisphere.

Underlying cardiogenic shock, cardiac arrest, and ARDS may predispose the brain to hypoxic-ischemic brain injury (HIBI) before ECMO is initiated, which can prime the brain for secondary injury (Inoue et al., 2020). Cerebrovascular dysregulation is a feature of HIBI (Sundgreen et al., 2001) and, in the setting of asymmetric ECMO blood flow, may lead to interhemispheric differences in CBF due to impaired cerebral autoregulation. In the setting of disrupted cerebral autoregulation post-arrest, optimal MAPs for cerebral perfusion are often higher than the clinically accepted norm of 65 mmHg (Sundgreen et al., 2001; Peberdy et al., 2010; Sekhon et al., 2019). Furthermore, interhemispheric communication may be disrupted resulting in regional independence of function and thus BFI oscillations that are independent on each side of the brain. Disruption in interhemispheric communication is seen in patients with traumatic brain and concussion (Schmidt et al., 2021; Wang et al., 2021), and thus may play a role in other global brain injuries such as HIBI. In addition, asymmetry in the intrinsic ischemic damage may result in differential dysregulation and cerebrovascular coupling, leading to asymmetry in the cerebral metabolic rate of oxygen consumption (CMRO_2_) and thus oxygen extraction fraction and local CO_2_ production. CO_2_ vasodilates cerebral arterioles and precapillary sphincters by increasing local [H^+^], triggering voltage-gated [K^+^] channels to hyperpolarize endothelial cells leading to vascular relaxation (Battisti-Charbonney et al., 2011). There are many other contributing factors affecting diameter of the blood vessels, including reduced cerebrovascular reactivity to vasomotor stimuli (Girouard and Iadecola, 2006), reduced CMRO_2_ in regions of cell death (Duckrow et al., 1981) and reduced CBF in proportion to neural activity (and thus CMRO_2_) (Bundo et al., 2002). Augmented by asymmetric perfusion resulting from the ECMO circuit, these differences may become even more pronounced as noted in our comatose patient cohort.

Our data showed potential for an increased sensitivity to the effects of post-oxygenator pH and pCO_2_ on rBF asymmetry with significant differences in linear model slopes between comatose and non-comatose patients. However, the associations themselves were not found to be significant. This discrepancy is likely due to low degrees of freedom secondary to small sample sizes when assessing each individual association’s significance, vs. increased degrees of freedom when comparing the two models against one another. ABG pH and pCO_2_ obtained from the patient’s right radial artery, representing native circulation, were not correlated with ASYM_rBF_, and importantly, nor were any differences noted between the two subgroup models. Of note, comatose patients had a higher, though nonsignificant, difference in PCO_2_ between pre- and post-oxygenator blood (Supplementary Table 1), implicating the degree of gas exchange in our findings. One possible reason for comatose patients having increased sensitivity to post-oxygenator pH and pCO_2_ is that these patients are more likely to be outside the zone of cerebral autoregulation (Van Den Brule et al., 2018). As implied above, it is possible that the left hemisphere is being exposed to the post-oxygenator blood more than the right side, and thus the effects of pCO_2_ on brain perfusion and autoregulation may be asymmetric with the difference being highlighted by an unstable neurovascular unit. While this evidence is insufficient to claim that differential association exists, it warrants further investigation. Should this finding hold true in larger future studies, it could provide novel evidence that post-oxygenator parameters should be controlled more tightly and in a more individualized manner, particularly for patients with suspected brain injury.

Previous studies have measured cerebral autoregulation using cross-correlation analysis between continuously measured blood pressure and either invasive (intracranial pressure, brain tissue oximetry) or non-invasive (NIRS-based cerebral oximetry, TCD-based CBF velocity) (Zweifel et al., 2008; Aries et al., 2010; Andresen et al., 2018; Montgomery et al., 2020) neuromonitoring modalities. We did not use this method because MAP variation was low (<5 mmHg) for long periods during monitoring, thus did not yield wide enough MAP ranges to generate full autoregulation curves. We did not utilize a circuit manipulation protocol to challenge MAP as has been performed in other studies (Papademetriou et al., 2012; Busch et al., 2020) because of the possible danger of secondary brain injury from hypo- or hyperperfusion. Though our methodology was unable to explore the upper and lower limits of autoregulation for each patient, optimal MAPs that minimized asymmetry can be identified in Supplementary Figure 4. The variability of this optimal MAP may represent the customized ranges of individual blood pressure targets for optimal cerebral perfusion observed in ECMO patients with HIBI (Rikhraj et al., 2021). Past clinical trials of increased MAP goals for cardiac arrest survivors resulted in improved cerebral oxygenation, but not improved outcomes (Jakkula et al., 2018; Ameloot et al., 2019). However, none of these studies utilized individualized, autoregulation-based MAP targets (Russo et al., 2017, 2018). The hypothesized benefit of CBF augmentation in this population is predicated on patchy hypoperfusion due to the no-reflow pathophysiology (Kloner et al., 2018). In contrast, the physiology by which asymmetric dysregulation may occur in our cohort remains to be elucidated.

Several limitations to our study must be considered when interpreting these data. Our study captured a small population with heterogeneous pathologies ranging from cardiac arrest to acute heart or lung failure. Not all patients were treated with the same modality of ECMO, and the physiology of VA ECMO differs significantly from VV ECMO as the latter relies on the native heart for forward uniform circulation. We analyzed ASYM_rBF_ in comatose vs. non-comatose patients excluding VV ECMO recipients in order to evaluate the effect of ECMO type on our findings and found comatose patients still had higher ASYM_rBF_ (*n* = 10, 28.7 ± 8.2 vs. 10.2 ± 2.5, two-tailed *p* = 0.04). We chose to keep VV ECMO patients in our study design as comparators with *a priori* risk of ischemic injury. Subjects varied by other factors such as cardiac function, level of sedation, receipt of NMB, and severity of illness (e.g., pre-ECMO SOFA score). Our small sample size precluded the ability to analyze these factors as covariates for association between ASYM_rBF_ and GCS-M. Furthermore, our monitoring methodology was not equipped to differentiate the source of cerebral perfusion (ECMO circuit vs. native circulation). Future studies should include specific ECMO populations with a single ECMO subtype and similar etiology necessitating ECMO.

One challenge we faced in designing our study was the need to dichotomize patients into “comatose” vs. “non-comatose” groups. Fundamentally, coma is a disordered state of consciousness in which the individual has no interaction with their environment (Provencio et al., 2020). A robust clinical definition of coma in the acute setting has not yet been developed and has often been disease-specific in prior research (Provencio et al., 2020). We relied on prior clinical trials (Nielsen et al., 2013; Lascarrou et al., 2019; Dankiewicz et al., 2021), to establish parameters for cohort dichotomization.

To derive rBF, the median BFI value was defined as the baseline for each monitoring session as was described in the method section. However, previous DCS studies define baseline differently (Durduran et al., 2009; Kim et al., 2010; Busch et al., 2019). In these studies, the baseline BFI is defined as a period of the measurement where the subject is at rest. This period of rest precedes a defined perturbation, allowing comparison of the relative change of BFI between the baseline and perturbation. Since this study did not include controlled perturbations, defining such a baseline period for these subjects is difficult and arbitrary. Additionally, there is no indication that the selected baseline region corresponds to when the subject is truly at rest, thus introducing user selection bias. We did not observe statistical significance in |ASYM_rBF_| between the two groups when using this method of normalization.

Alternatively, the mean value of the BFI time series could be used as a baseline for calculating rBF (Giovannella et al., 2020) but this would be disproportionately affected if there are spontaneous changes in BFI. The median is less affected by such changes than the mean. We compared the median with the mean resting baseline values calculated in the following manner. First, each monitoring period was smoothed using a moving average filter and regions where drastic flow changes occurred were removed. This modified signal was then averaged to define a baseline to calculate rBF. The difference between the median and the mean resting baseline varied case-by-case (i.e., sometimes they matched well, and other times they differed significantly). Despite the difference, ASYM_rBF_ computed using the mean resting baseline for normalization showed statistical significance between the two groups (comatose vs. non-comatose) as was shown using the median value (*p* < 0.006 for one-tailed and *p* < 0.01 for two-tailed). This affirms that using the median value as the baseline definition did not alter the major findings of this study. This might be due to our data having relatively few incidents of drastic changes in BFI as many patients were sedated or kept stable. While this mean resting baseline method seems to capture the baseline well, it does require user input when deciding on regions of drastic BFI changes. Thus, we chose to use the median as the baseline to allow for consistent selection for all the measurement periods. Future studies will continue to investigate the optimal method to estimate baseline values.

Measuring blood flow with DCS also presents its own set of limitations. As with all near-infrared spectroscopic methods, DCS is limited in its depth of penetration to cortical tissue (Buckley et al., 2014). Blood flow in subcortical regions is not well-captured. Moreover, spatial resolution was limited to the forehead because DCS is much more sensitive to interference from hair than continuous wave NIRS, which has been employed with multiple detectors throughout the head in other studies (Papademetriou et al., 2013). The lack of spatial resolution is less likely to be a limitation in whole-brain injury, including states of reduced consciousness or coma, as the entire hemisphere may be affected to a similar magnitude (Kaloostian et al., 2012; Ohshima et al., 2012; Foley et al., 2013; Sato et al., 2014; Baker et al., 2019).

Our simultaneous measurements of CBFV with TCD enabled us to compare the asymmetry in CBF measured by our experimental device vs. clinical gold standard. Using Bland–Altman analysis, we found that the two methods did not significantly differ in their measurement of asymmetry and thus lent further support to the validity of DCS measurements. However, it must be noted that this comparison was done over a range of MAP values limited to those the patient experienced during simultaneous TCD and DCS measurement, a limitation of manually placed TCD probe measurement. Future use of either phased array Doppler ultrasound (Pietrangelo et al., 2018) or headframe-assisted probe placement would facilitate longer-term measurements and enable a more robust comparison between DCS and TCD measurements of CBF.

Another limitation of our DCS measurements was the use of constant optical coefficients of scattering and absorption. These coefficients were based on data in the literature (Brady et al., 2010; Parthasarathy et al., 2018; Selb et al., 2018). Incorrect values can lead to error in the BFI calculation, particularly the scattering coefficient. It is reasonable to assume the scattering coefficient does not change during brain monitoring. However, absorption may change based on the changes in total hemoglobin concentration or blood oxygen saturation. Relative changes in blood flow are less sensitive to absorption changes, compared to absolute BFI values. Our results remain valid since we are quantifying the difference between hemispheres based on relative changes in blood flow. Nevertheless, concurrently quantifying absorption and scattering coefficients will improve the accuracy of the BFI. In the future, a frequency domain NIRS system will be added to extract coefficients of absorption and scattering alongside DCS measurements.

The manner in which DCS was utilized in this study does not allow for measurements of absolute blood flow, but instead uses a relative measure of CBF. Our asymmetry measure is based on the rBF normalized to the median value for each day, which allows us to better compare between days and subjects. Absolute blood flow calibration can require imaging modalities such as CT perfusion (Verdecchia et al., 2016) or MRI arterial spin labeling (Durduran et al., 2004; Carp et al., 2010). We decided against using these techniques for this pilot study to minimize transport to CT scanners and avoid nephrotoxic exposure to intravenous contrast dye; furthermore, the ECMO circuit is not MRI compatible. A recent study utilized time-resolved dynamic contrast enhanced NIRS (DCE-NIRS) in conjunction with a one-time bolus of indocyanine green to calibrate DCS measurements in critically ill adults (He et al., 2018). The use of indocyanine green may be contraindicated in patients with renal dysfunction, which is common among patients with severe cardiac disease (Hu et al., 2016; Sandroni et al., 2016). Nevertheless, absolute CBF calibration may be important in quantifying differences in CBF as the cause of asymmetric perfusion.

## Conclusion

In this study of adult ECMO recipients, comatose patients demonstrated increased cerebral hemispheric perfusion asymmetry as measured with DCS when compared to non-comatose patients. Future studies will include larger sample sizes, other modalities of neuromonitoring, and patients with specific conditions to validate hemispheric asymmetry as a biomarker of neuronal dysfunction in this population. Ultimately, it remains to be seen whether this marker is a predictor of coma recovery, informs pathophysiological basis of asymmetric perfusion, and provides a goal for targeted ECMO therapy.

## Data Availability Statement

The raw data supporting the conclusions of this article will be made available by the authors, without undue reservation.

## Ethics Statement

The studies involving human participants were reviewed and approved by the University of Rochester Research Subjects Review Board. The patients/participants provided their written informed consent to participate in this study.

## Author Contributions

TJ contributed to study concept and acquisition, analysis, and interpretation of the data, and played a significant role in drafting this manuscript. ID contributed to study design and acquisition, analysis, and interpretation of the data, and played a significant role in drafting this manuscript. KD, YX, and ES contributed to data acquisition and analysis. OS contributed to study concept and design, participated in patient recruitment, as well as data interpretation, and revised this manuscript for intellectual content. MM and RM contributed to study concept and design, as well as data interpretation, and revised this manuscript for intellectual content. TW contributed to statistical analysis. IG contributed to data acquisition and study design, and revised this manuscript for intellectual content. GS contributed to study concept and design, and revised this manuscript for intellectual content. RC contributed to study concept, design, data acquisition, analysis and interpretation, and revised this manuscript for intellectual content. IK contributed to study design, concept, data acquisition, interpretation, and drafted and revised this manuscript. All authors contributed to the article and approved the submitted version.

## Conflict of Interest

The authors declare that the research was conducted in the absence of any commercial or financial relationships that could be construed as a potential conflict of interest.

## Publisher’s Note

All claims expressed in this article are solely those of the authors and do not necessarily represent those of their affiliated organizations, or those of the publisher, the editors and the reviewers. Any product that may be evaluated in this article, or claim that may be made by its manufacturer, is not guaranteed or endorsed by the publisher.
