# Supplementary material for: Cerebral Blood Flow Hemispheric Asymmetry in Comatose Adults Receiving Extracorporeal Membrane Oxygenation
Source: Front Neurosci. 2022 Apr 11;16:858404. doi: 10.3389/fnins.2022.858404 (PMC9036108; doi:10.3389/fnins.2022.858404)
Supplement: Supplementary file 1 [file Data_Sheet_1.docx]

Cerebral Blood Flow Hemispheric Asymmetry in Comatose Adults Receiving Extracorporeal Membrane Oxygenation

Supplementary Material


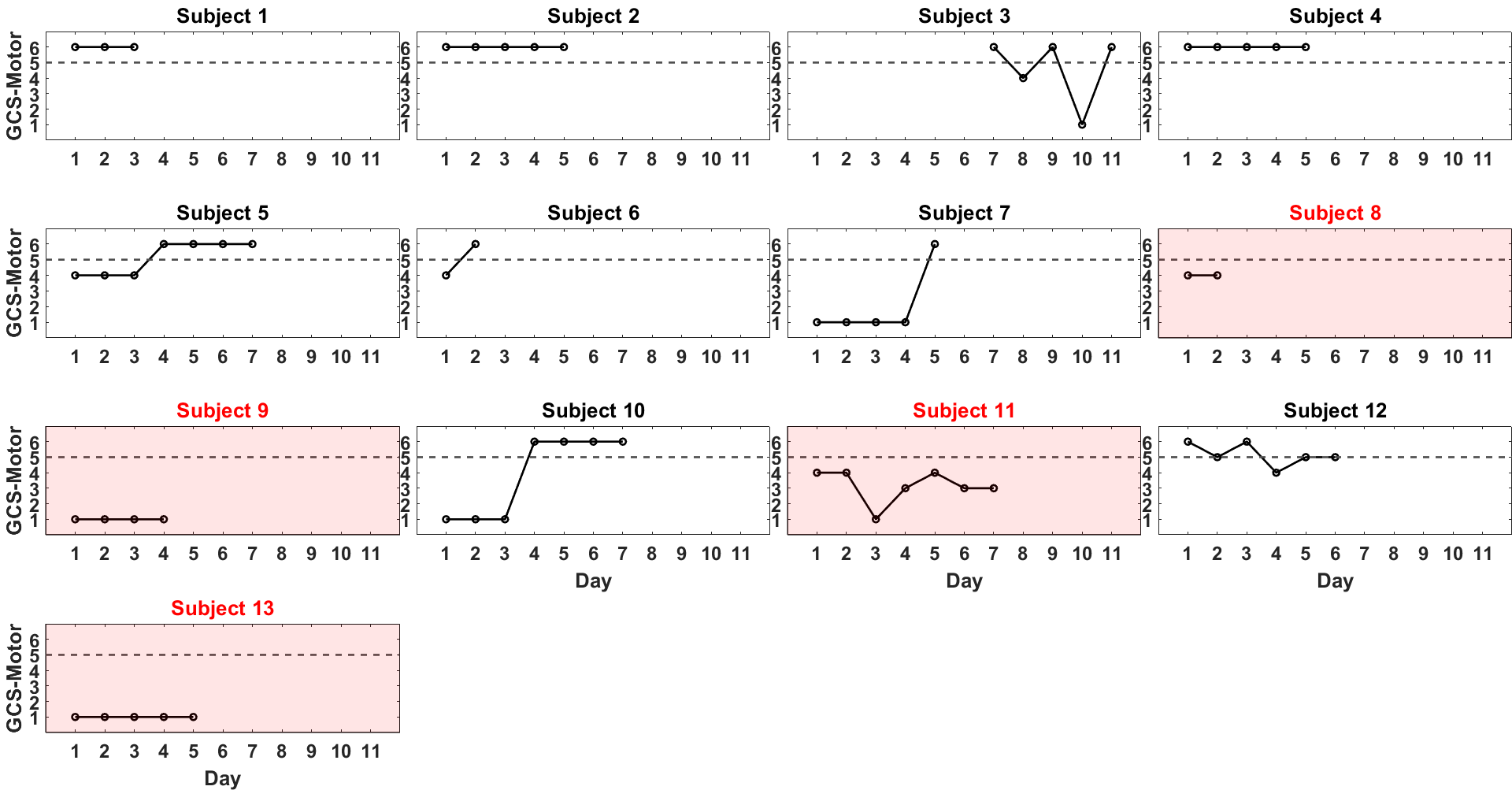


Figure S1: GCS-Motor scores for all the patients for each day of their monitoring. Dashed line indicates a GCS-Motor score of 5, the threshold of whether a patient is non-comatose. Subjects 7 and 10 had low GCS-Motor scores on the first days of monitoring due to neuromuscular blockade. GCS data for the first 6 days of monitoring for subject 3 are not reported since ASYM_rBF_ was not calculated for those days. For subjects 8, 9, 11, and 13 which are highlighted in red, their GCS-Motor score never increased above 4, and thus were defined as comatose.


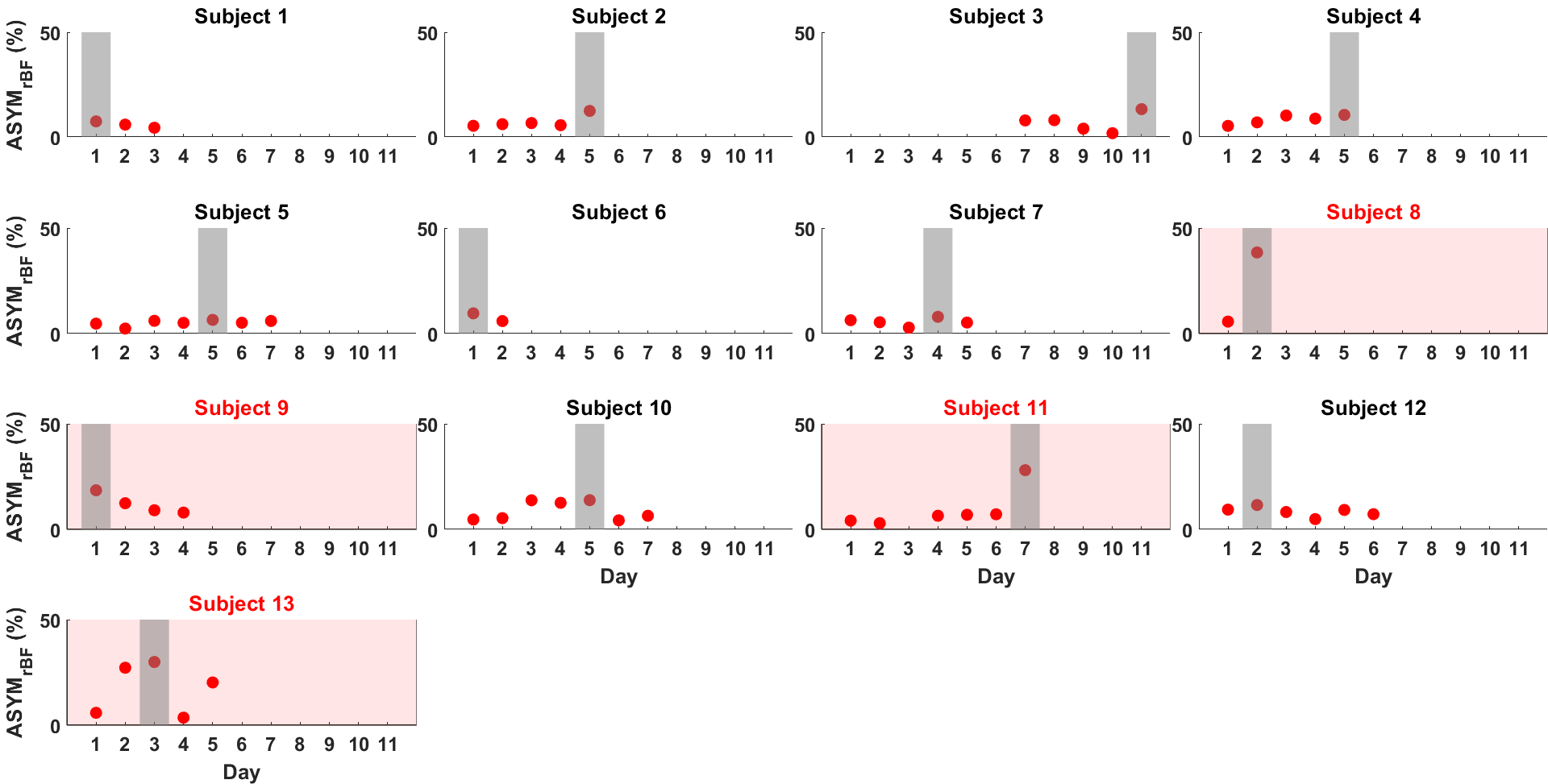


Figure S2: ASYM_rBF_ (red) plotted for all patients during their monitoring period. The shaded region denotes the day ASYM_rBF_ was the maximum for each subject. Data from Subject 3 days 1 through 6 and subject 11 day 3 were discarded due to low SNR. Subjects highlighted in red were subjects that were defined as comatose during ECMO treatment.


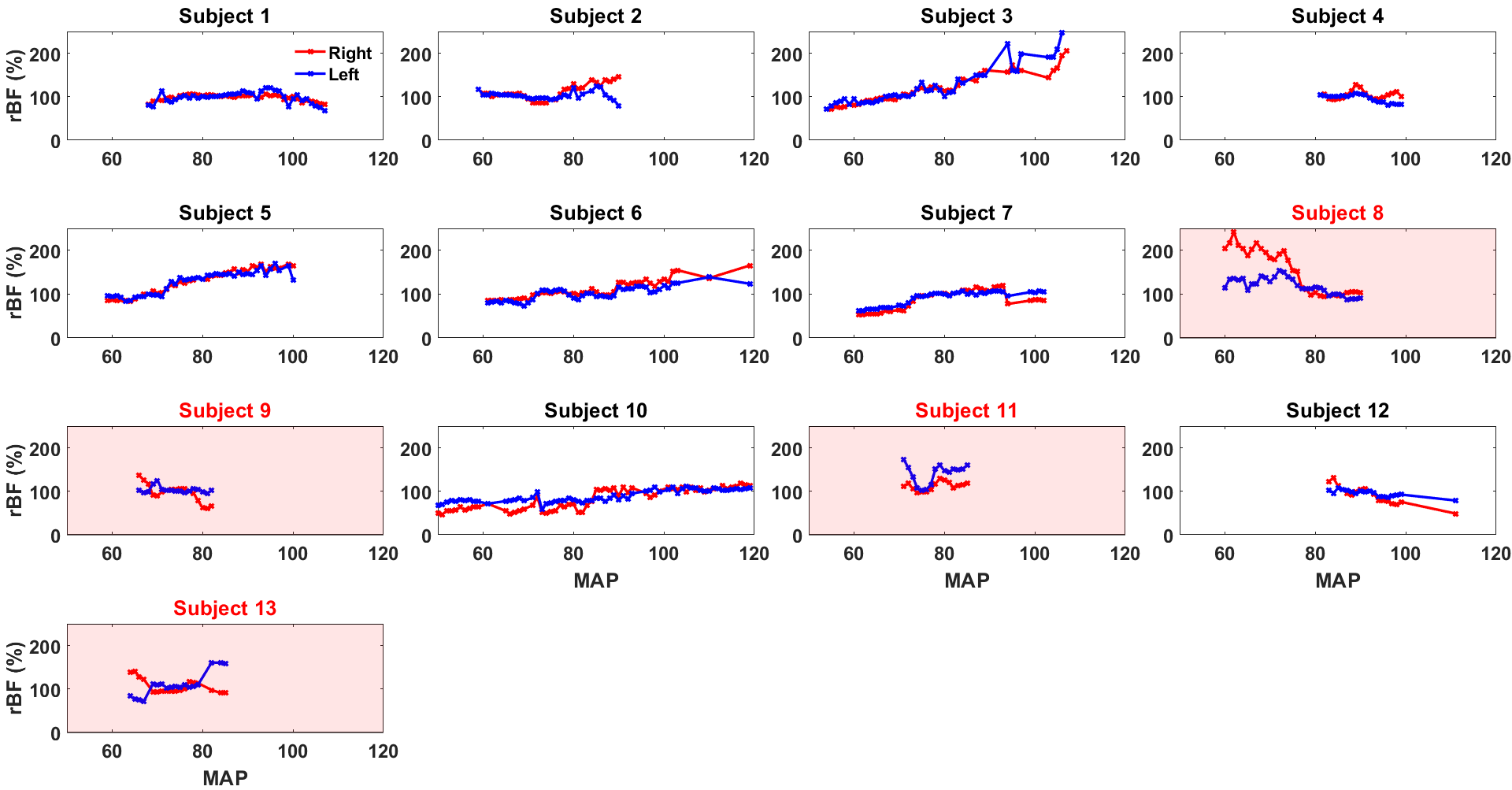


**Figure S3: Relative blood flow (rBF) of the right hemisphere (red) and left hemisphere (blue) plotted against mean arterial pressure (MAP) for all patients on the day that exhibited maximum ASYM_rBF_. Comatose patients are highlighted in red.**


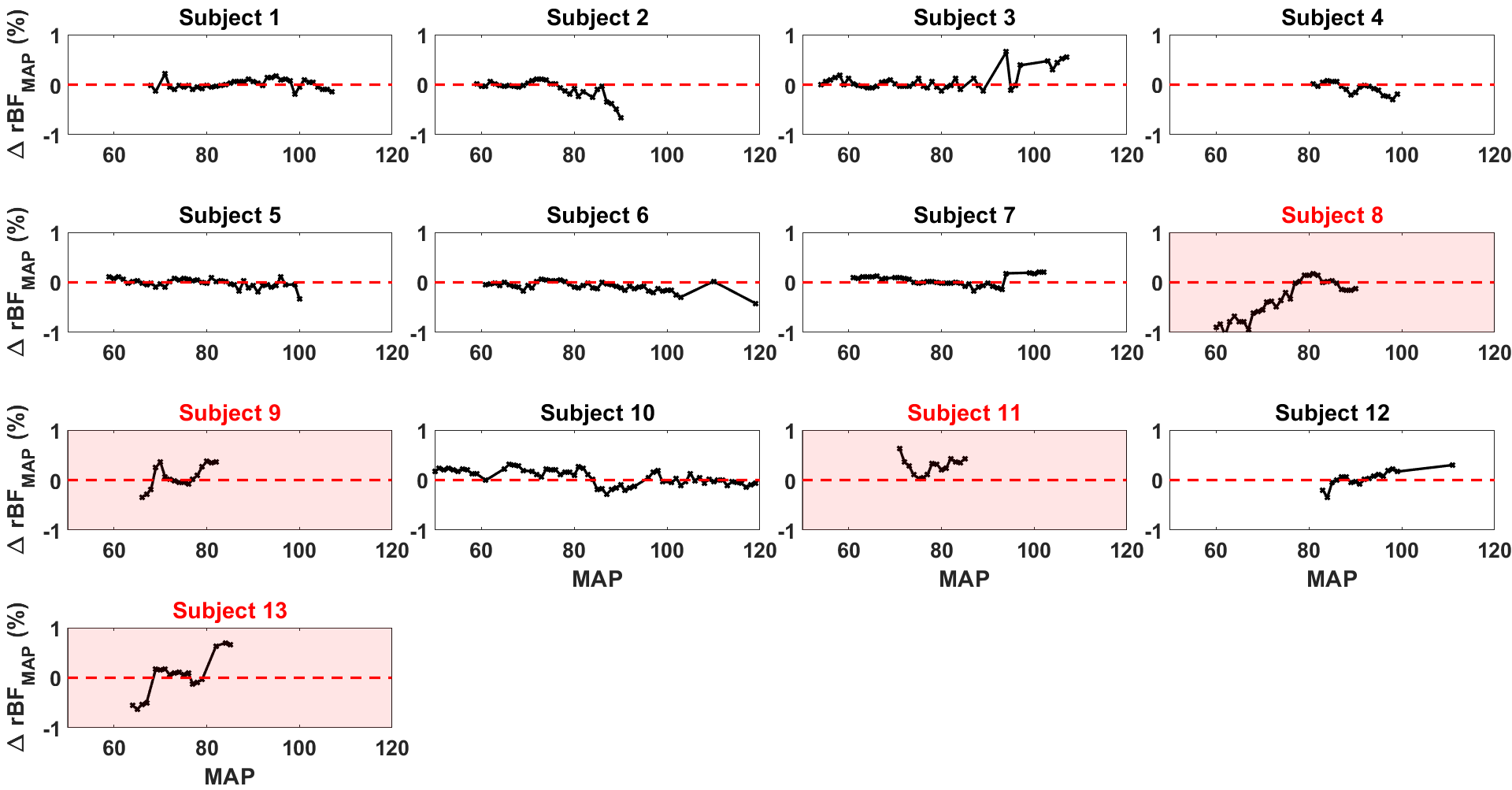


Figure S4: ΔrBF_MAP_ graph of all subjects. Subjects (8, 9, 11, and 13) were defined as comatose and highlighted in red. Red dotted line indicates a ΔrBF_MAP_ value of zero (symmetric rBF). Negative ΔrBF_MAP_ values indicate right hemisphere rBF > left hemisphere.

Figure S5: Group summary of TCD and DCS interhemispheric cerebral blood flow asymmetry measurement comparison. The difference between asymmetry measurements, averaged over each data collection sessions during which both TCD and DCS data were collected, is shown for each individual subject as indicated by the circle color legend. The group median was -0.32 [IQR -1.56-4.01].

| Clinical Variable^1^ | Non-Comatose | Comatose | P-Value |
| --- | --- | --- | --- |
| Monitoring duration (hrs) | 2.2 [1.3] | 2.3 [0.7] | 0.58 |
| ABG pH | 7.46 ± 0.04 | 7.49 ± 0.04 | 0.30 |
| ABG pO2 | 88 [120.8] | 179.5 [143] | 0.28 |
| ABG pCO2 | 35 [3.8] | 35.5 [12] | 1.00 |
| ABG Lactate | 1.5 ± 0.9 | 1.9 ± 1.3 | 0.55 |
| Pre-Oxy pH | 7.39 [0.07] | 7.4 [0.09] | 0.81 |
| Pre-Oxy pO2 | 46.4 ± 13.5 | 48 ± 4.3 | 0.83 |
| Pre-Oxy pCO2 | 43.7 ± 11.4 | 49 ± 9.9 | 0.44 |
| Post-Oxy pH | 7.43 ± 0.06 | 7.44 ± 0.07 | 0.88 |
| Post-Oxy pO2 | 440.2 ± 80.6 | 420.3 ± 48.8 | 0.66 |
| Post-Oxy pCO2 | 39.2 ± 8.5 | 40.3 ± 9.9 | 0.85 |
| Post-ABG pH | -0.02 ± 0.05 | -0.04 ± 0.10 | 0.59 |
| Post-ABG pO2 | 293.0 ± 133.4 | 231.3 ± 89.4 | 0.42 |
| Post-ABG pCO2 | 1.7 ± 6.7 | 4.8 ± 10.2 | 0.53 |
| Post-Pre pH | 0.04 [0.02] | 0.08 [0.04] | 0.05 |
| Post-Pre pO2 | 393.8 ± 69.9 | 372.3 ± 46.2 | 0.59 |
| Post-Pre pCO2 | -5 [4.30] | -9 [3.5] | 0.08 |
| SOFA | 12 [5.3] | 12 [9] | 0.92 |
| MAP | 80.7 ± 9.3 | 76.2 ± 4 | 0.38 |
| MAP Range | 60.3 ± 18.7 | 42.3 ± 21.3 | 0.15 |
| Pulse Pressure | 45.7 ± 19.7 | 39.6 ± 50.2 | 0.83 |
| Pulse Pressure Range | 46.0 ± 18.2 | 19.6 ± 10.4 | 0.02 |
| ECMO Flow^2^ | 1.5 [0.5] | 1.7 [1.2] | 0.57 |

Table S1: Average value of clinical characteristics for comatose and non-comatose patients for the day of maximum ASYM_rBF_. ABG: Arterial blood gas from right radial artery. Pre- and Post-Oxy indicate blood drawn from pre- and post-oxygenator cannulas of the ECMO circuit. Post-ABG and post-pre denotes difference in values between post-oxygenator blood, ABG, and pre-oxygenator blood. ^1^Lists mean ± standard deviation for normally-distributed variables and median [interquartile range] for non-parametric variables. ^2^ECMO flow in liters per minute normalized for body surface area.

| GCS-M score | Description |
| --- | --- |
| 1 | No movement |
| 2 | Extensor reflex |
| 3 | Flexor reflex |
| 4 | Withdraws from pain |
| 5 | Localizes to pain |
| 6 | Follows commands |

Table S2. Glasgow Coma Scale motor subscores (GCS-M score). Adapted from Teasdale and Jennett, 1974 (Teasdale and Jennett, 1974).
